# Widespread marine and freshwater distributions of active sulfoquinovose-degrading bacteria

**DOI:** 10.1093/ismejo/wrag155

**Published:** 2026-06-17

**Authors:** Guohua Liu, Xuanyun Qiu, Rongguang Cao, Changjie Dong, Quanrui Chen, Shujing Liu, Wenhao Li, Xuejing Li, Nianzhi Jiao, Spencer J Williams, Yao Zhang, Kai Tang

**Affiliations:** State Key Laboratory of Marine Environmental Science, College of Ocean and Earth Sciences, Fujian Key Laboratory of Marine Carbon Sequestration, Xiamen University, Xiamen 361102, China; State Key Laboratory of Marine Environmental Science, College of Ocean and Earth Sciences, Fujian Key Laboratory of Marine Carbon Sequestration, Xiamen University, Xiamen 361102, China; State Key Laboratory of Marine Environmental Science, College of Ocean and Earth Sciences, Fujian Key Laboratory of Marine Carbon Sequestration, Xiamen University, Xiamen 361102, China; State Key Laboratory of Marine Environmental Science, College of Ocean and Earth Sciences, Fujian Key Laboratory of Marine Carbon Sequestration, Xiamen University, Xiamen 361102, China; State Key Laboratory of Marine Environmental Science, College of Ocean and Earth Sciences, Fujian Key Laboratory of Marine Carbon Sequestration, Xiamen University, Xiamen 361102, China; State Key Laboratory of Marine Environmental Science, College of Ocean and Earth Sciences, Fujian Key Laboratory of Marine Carbon Sequestration, Xiamen University, Xiamen 361102, China; State Key Laboratory of Marine Environmental Science, College of Ocean and Earth Sciences, Fujian Key Laboratory of Marine Carbon Sequestration, Xiamen University, Xiamen 361102, China; State Key Laboratory of Marine Environmental Science, College of Ocean and Earth Sciences, Fujian Key Laboratory of Marine Carbon Sequestration, Xiamen University, Xiamen 361102, China; State Key Laboratory of Marine Environmental Science, College of Ocean and Earth Sciences, Fujian Key Laboratory of Marine Carbon Sequestration, Xiamen University, Xiamen 361102, China; School of Chemistry and Bio21 Molecular Science and Biotechnology Institute, University of Melbourne, Parkville, Victoria 3010, Australia; State Key Laboratory of Marine Environmental Science, College of Ocean and Earth Sciences, Fujian Key Laboratory of Marine Carbon Sequestration, Xiamen University, Xiamen 361102, China; State Key Laboratory of Marine Environmental Science, College of Ocean and Earth Sciences, Fujian Key Laboratory of Marine Carbon Sequestration, Xiamen University, Xiamen 361102, China

**Keywords:** DNA-stable isotope probing, sulfoquinovose-degrading bacteria, sulfur cycle, aquatic environments

## Abstract

Sulfoquinovose (SQ), a sulfonated sugar produced on a gigaton scale each year, contributes to global sulfur cycling, yet the microbes and pathways mediating its turnover in the environment have been inferred largely from genomic potential rather than direct activity. Here, we coupled incubations of environmental samples with ^13^C-labeled SQ to DNA-stable isotope probing to identify active SQ carbon assimilators across estuary, mangrove, and lake ecosystems. In estuarine communities, *Vibrio* and *Cognatishimia* incorporated SQ-derived ^13^C; *Novosphingobium* dominated in the mangrove, and *Agrobacterium* in the lake. Pure-culture experiments, coupled with comparative proteomics and gene knockout validation, demonstrated that *Vibrio* strains degrade SQ via modified sulfoglycolytic Embden–Meyerhof–Parnas and Entner–Doudoroff pathways to produce the environmentally significant organosulfur 2,3-dihydroxypropanesulfonate. Comparative genomic analyses suggested that closely related genome representatives of *Novosphingobium, Cognatishimia*, and *Agrobacterium* encode the sulfolytic SQ monooxygenase pathway. A global survey of aquatic microbial genomes indicated that over 9% harbor SQ degradation clusters, supporting a widespread distribution of bacterial SQ catabolic potential in aquatic environments.

## Graphical abstract

**Graphical Abstract:**
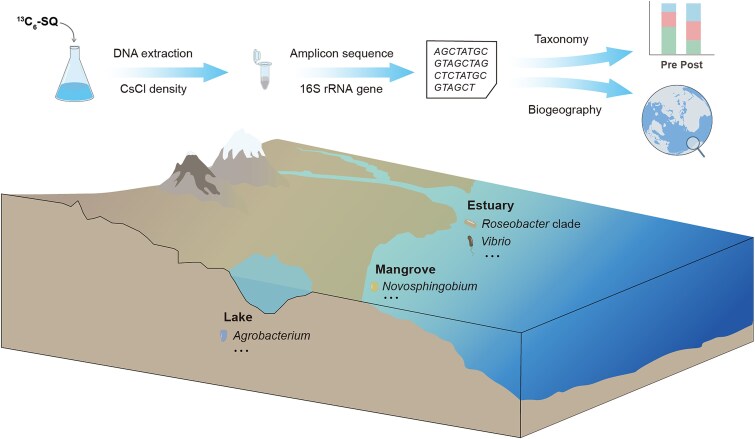

## Introduction

The global sulfur cycle is sustained by a suite of organosulfur metabolites produced in immense quantities by living systems, among which sulfoquinovose (SQ) and its conjugates stand out as among the most abundant. SQ occurs as the polar head group of sulfoquinovosyl diacylglycerol (SQDG), a sulfolipid ubiquitous in the photosynthetic membranes of plants, algae, and phototrophic bacteria [1], where it contributes to structural integrity, including under environmental stress and phosphorus deficiency [2]. With an estimated annual production of billions of tons carbon equivalent per year [3], SQ represents a major component of the organosulfur pool in the biosphere [3–5]. Yet, despite its abundance, the microbial lineages responsible for SQ degradation in environmental systems, and the pathways they deploy remain poorly resolved.

In marine ecosystems, research on SQ has focused primarily on phytoplankton sources, where it is synthesized by diatoms, cyanobacteria, and green and red algae [3, 6, 7]. SQ is released into seawater through cellular exudation and viral lysis, contributing an estimated 1.3 teragrams annually to global marine production, calculated on a molecule-of-SQ basis rather than C or S normalized units [6]. Once released, algal-derived SQ forms a trophic interface and metabolic currency, mediating carbon–sulfur exchanges between primary producers and heterotrophic bacteria [8]. Field measurements in the Bohai Sea report SQ concentrations averaging 0.88 ± 0.20 μmol g^−1^ particulate organic carbon [6]. However, the biogeochemistry of SQ is largely unexplored in other aquatic ecosystems, including mangroves, lakes, and estuaries.

Liberation of free SQ from SQDG, or its delipidated derivative sulfoquinovosyl glycerol (SQGro), is catalyzed by sulfoquinovosidases from glycoside hydrolase families 31 and 188 [9, 10]. Multiple bacterial pathways for SQ catabolism have now been described [11–17]. Two aerobic routes, the sulfoquinovose monooxygenase (sulfo-SMO) and sulfoquinovose dioxygenase (sulfo-SDO) pathways, oxidatively cleave SQ to yield sulfite and 6-dehydroglucose, which is subsequently reduced to glucose [15, 17]. The sulfo-SMO pathway is prevalent in rhizobial strains and *Roseobacter* clade bacteria [6, 15], whereas the sulfo-SDO pathway is characteristic of *Oceanospirillaceae* and *Alteromonadaceae* within the *Gammaproteobacteria* [3, 17]. In contrast, sulfoglycolytic pathways convert SQ into short-chain organosulfonates such as sulfolactate (SL), 2,3-dihydroxypropanesulfonate (DHPS), sulfoacetate, and isethionate, which may be further transformed into compounds such as sulfopropionate and 3-hydroxypropanesulfonate, before being secreted and undergoing cross-feeding for complete mineralization to sulfate or H_2_S [18–20]. For example, the sulfo-EMP, sulfo-ED and sulfofructose-transaldolase (sulfo-TAL) pathways produce the intermediate sulfolactaldehyde, which may be oxidized to SL or reduced to DHPS [11–14], and the sulfo-transketolase (sulfo-TK) pathway produces sulfoacetaldehyde, which may be oxidized to sulfoacetate or reduced to isethionate [14, 16].

Although the SQ degradation pathways have been defined in cultured bacterial isolates [9–13, 15, 17, 21–23], the diversity and ecological roles of SQ-degrading microorganisms in natural environments remain poorly understood. So far, studies have been confined to the human and mouse gut microbiomes [18, 24], and to seawater [6]. DNA-stable isotope probing (DNA-SIP) technology links substrate assimilation to phylogeny, enabling the identification of microorganisms assimilating SQ-derived carbon under defined incubation conditions. Coupled with comparative genomics using curated resources such as the proGenomes database [25], it resolves both taxonomic breadth and pathway usage, defining which microbes degrade SQ and how.

In this study, we systematically quantified SQ concentrations across salinity gradients in estuarine, mangrove forest, and freshwater lake environments. Microcosm incubations with ^13^C-labeled SQ coupled to DNA-SIP were used to identify active SQ-assimilating taxa in these ecosystems. Subsequent genomic analyses resolved the phylogenetic distribution and pathway diversity of bacterial SQ catabolism. Together, these approaches bridge pathway knowledge derived from cultured isolates with the poorly understood ecology of SQ degradation in natural environments. By linking SQ-amended microcosm incubations, physiological validation in isolated strains, and comparative genomics, we provide evidence for the diversity, activity, and environmental distribution of SQ-degrading bacteria across selected aquatic ecosystems.

## Materials and methods

### Incubation of environmental samples with ^13^C_6_-SQ

Twenty-liter water samples were collected at each of five stations spanning the Jiulong River estuary (JR), the Shenzhen Futian mangrove forest (MF), and Furong Lake (FRL) (Fig. S1). *In situ* water parameters, including temperature, salinity, and pH, were measured following established protocols [26], and total bacterial abundance was determined using an Accuri C6 flow cytometer (BD Biosciences) after staining cells with SYBR Green I. All samples were first passed through a 20 μm nylon screen to remove algae, large particles, and debris. A portion of each sample was retained for direct measurement of SQ concentrations, and the remainder was precultured in the dark at 25°C for 48 h to prepare for microcosm experiments.

To distinguish particle-associated (PA) and free-living (FL) bacterial communities, water samples were size fractionated by filtration. FL communities were obtained by filtration of samples through 3-μm polycarbonate membranes (47 mm, Millipore, USA), yielding groups JR1-FL, JR2-FL, JR3-FL, MF-FL, and FRL-FL. The corresponding PA fractions (JR1-PA, JR2-PA, JR3-PA, and MF-PA) were retained on 3-μm membranes and resuspended in water that had been filtered through 0.2-μm polycarbonate membranes (47 mm, Millipore, USA) from their respective stations.

For isotope tracing, ^13^C_6_-SQ was synthesized from ^13^C_6_-glucose using a previously published method [27]. Each treatment was set up in triplicate and incubated in the dark at 25°C. For each experimental unit, 1.2 L of water sample for incubation was transferred into a pre-combusted 2-L glass bottle (450°C, 4 h). Experimental groups received a final concentration of 100 μM ^13^C_6_-SQ, whereas controls received 100 μM unlabeled SQ (^12^C_6_-SQ). Bottles were sealed with sterile gas-permeable membranes (0.2–0.3 μm pore size, 16 mm diameter, BKMAMLAB) and incubated in the dark at 25°C with shaking (50 rpm; triplicates per treatment). Subsamples were collected at 0, 24, 48, 72, and 84 h to monitor SQ concentrations. Microbial communities were harvested by filtration through 0.2-μm polycarbonate membranes (47 mm, Millipore, USA) at the start (0 h) and end (84 h) of incubation, flash-frozen in liquid nitrogen, and stored at −80°C.

### Quantitative determination of sulfoquinovose and 2,3-dihydroxypropanesulfonate concentrations

SQ concentrations in microcosm incubation samples, isolated bacterial cultures, and environmental extracts, as well as DHPS concentrations in isolated bacterial cultures, were quantified using ultra-high-performance liquid chromatography coupled with mass spectrometry (UHPLC–MS). Analyses were performed on an Agilent 1290 Infinity II UHPLC system coupled to an Agilent 6460 triple quadrupole mass spectrometer (Agilent, CA, USA), fitted with a BEH Amide column (1.7 μm, 2.1 × 100 mm; Waters, MA, USA). The mobile phases consisted of solvent A (100% acetonitrile) and solvent B (Milli-Q water containing 10 mM ammonium acetate). The column was equilibrated with 75% A/25% B for 10 min prior to injection. The gradient profile was as follows: 25%–50% B over 8.5 min, holding at 50% B for 1 min, reversion to 25% B over 0.5 min, and holding at 25% B for 10 min. For mass spectrometric detection, the primary and secondary ion transitions were *m/z* 243 → 183 for ^12^C_6_-SQ, *m/z* 249 → 187 for ^13^C_6_-SQ, and *m/z* 155 → 80 for DHPS. Data acquisition and quantification were carried out using MassHunter Workstation Software (version B.08.00; Agilent Technologies, USA).

### DNA extraction and cesium chloride density gradient ultracentrifugation

Bacterial DNA retained on filter membranes was extracted using the FastDNA Spin Kit for Soil (MP Biomedicals, USA) in combination with bead-beating to enhance cell lysis efficiency. DNA concentration was determined with the Qubit dsDNA Assay Kit and Qubit 2.0 fluorometer (Invitrogen, USA). CsCl density gradient ultracentrifugation and fractionation were performed following published protocols with minor modifications [28, 29]. GB buffer (0.1 M Tris–HCl, 0.1 M KCl, 1 mM EDTA) was added to the CsCl solution, and the final density was adjusted to 1.725 g ml^−1^. For each treatment, equal amounts of DNA from three independently grown replicate cultures were pooled. Approximately 2 μg of DNA was mixed with 5.1 ml of CsCl solution (7.16 M) and transferred into Quick-Seal centrifuge tubes (Beckman Coulter, USA).

Centrifugation was carried out in an Optima L-100 XP ultracentrifuge (Beckman Coulter, CA, USA) equipped with a VTi 65.2 vertical tube rotor at 140 000 × *g*, 20°C, for 72 h. Following centrifugation, gradients were fractionated into 15 equal volumes (~320 μL each) using a syringe pump (Braintree Scientific, USA). Fraction densities were determined with a digital refractometer (Brix/RI Chek, Reichert) at 20°C. Fractions with buoyant densities >1.725 g ml^−1^ were classified as heavy DNA fractions, whereas fractions with densities <1.725 g ml^−1^ were classified as light DNA fractions (Fig. S2). DNA from each fraction was precipitated by adding two volumes of PEG solution (30% PEG 6000, w/v; 1.6 M NaCl; 20 μg glycogen), washed with 70% ethanol, air-dried, and resuspended in ultrapure water.

### Quantitative polymerase chain reaction of density fractions

The abundance of bacterial 16S rRNA genes in each fraction was quantified using quantitative polymerase chain reaction (qPCR) with Bac-338F and Bac-518R primers on a CFX 96 real-time instrument (BIO-RAD) [30]. Standard curves were constructed using the target DNA fragments of *Escherichia coli* strain P10 [31]. The PCR mixture contained 10 μL of SYBR Premix Ex Taq II, 5 μg of bovine serum albumin, 0.5 μM of each primer, and 1 μL of template DNA in a total volume of 20 μL. Each reaction mixture was subjected to qPCR in triplicate with the following program: initial enzyme activation at 95°C for 105 s, followed by 40 cycles of 95°C for 15 s, 55°C for 30 s, and 72°C for 30 s. Triplicate non-template reactions were included as negative controls during each run of the program.

### DNA sequencing and analysis

Total community DNA samples collected before and after incubation with ^12^C_6_-SQ or ^13^C_6_-SQ, together with selected density gradient fractions (5–10 fractions per sample, Fig. S2) chosen based on 16S rRNA gene abundance across the density gradient, were subjected to 16S rRNA gene amplicon sequencing. The V3–V4 hypervariable regions of the 16S rRNA gene were amplified using barcoded universal primers (343F and 798R, Table S1) on an ABI GeneAmp 9700 thermal cycler (Thermo Fisher Scientific, USA) [32]. PCR amplifications were performed in triplicate in 20 μL reaction mixtures containing 4 μL of 5× FastPfu Buffer, 2 μL of 2.5 mM dNTPs, 0.8 μL of each primer (5 μM), 0.4 μL of FastPfu Polymerase, and 10 ng of normalized template DNA. The PCR program consisted of an initial denaturation at 95°C for 2 min, followed by 25 cycles of 95°C for 30 s, 55°C for 30 s, and 72°C for 30 s, with a final extension at 72°C for 5 min. PCR products were visualized on 2% agarose gels, purified with the AxyPrep DNA Gel Extraction Kit (Axygen Biosciences, USA), and quantified with a Qubit 3.0 fluorometer (Invitrogen, USA). A total of 24 barcoded amplicons were pooled in equimolar amounts to construct a paired-end Illumina sequencing library. Sequencing was performed on a NovaSeq 6000 System (Illumina) at Shanghai Biozeron Biotechnology Co., Ltd. (Shanghai, China).

Raw sequencing data were quality-filtered and demultiplexed using Trimmomatic [33], with reads containing an average Phred quality score < 20 within a 10 bp sliding window discarded. High-quality sequences were processed through the QIIME2 pipeline [34], including read merging and chimera removal, to generate amplicon sequence variants (ASVs). Taxonomic assignment of ASVs was carried out using the UCLUST algorithm (https://github.com/topics/uclust) against the SILVA SSU database (version 138) [35], with a minimum confidence threshold of 80%.

After incubation with ^13^C_6_-SQ, an ASV was considered to have incorporated ^13^C if (i) its relative abundance in the heavy DNA-SIP fractions was significantly higher than that in the corresponding light fractions from the same incubation, the statistical analysis was performed using the Wilcoxon rank-sum tests implemented via the wilcox.test() function in the R software environment, and (ii) its peak 16S rRNA gene copy number shifted toward higher densities compared to the ^12^C_6_-SQ control. For each ASV (*i*), its absolute copy number (*y_i_*) in a given density fraction (*j*) was calculated by multiplying its relative 16S rRNA gene abundance in that fraction (*p_ij_*) by the total 16S rRNA gene copy number of the same fraction (*f_j_*). This allowed estimation of the absolute abundance of each ASV across the density gradient.

### Determination of ^13^C-labeling between unlabeled and labeled density fractions

The distribution of bacterial 16S rRNA gene abundance along the CsCl density gradient was assessed by combining fraction density measurements, qPCR-derived 16S rRNA gene copy numbers, and ASV-based 16S rRNA gene abundances. The densities of labeled (*W*_LABi_) and unlabeled (*W*_LIGHTi_) DNA for each taxon were determined by aggregating ASVs assigned to the same genus and normalizing to qPCR-derived copy numbers, following established methods [36]. To minimize bias from baseline variability, only the principal DNA peak was integrated; specifically, fractions containing >20% of the maximum observed abundance were included [37]. Because 16S rRNA gene copy numbers can vary by orders of magnitude across taxa and density fractions, maximum normalization was applied within each sample to standardize copy numbers, thereby enabling comparability of DNA density gradient profiles across taxa and fractions.

### Phylogenetic analyses of 16S rRNA gene sequences

Sequences of each ^13^C-labeled ASV were analyzed using BLASTn on the NCBI 16S rRNA database to identify their closest relatives. ASVs and available reference sequences were aligned using MAFFT (version 7.520) [38]. Maximum-likelihood phylogenies were reconstructed with IQ-TREE (version 2.0.3) [39] under the best-fit substitution model (TIM3 + F + I + G4). Node support was assessed by 1000 bootstrap replicates. Final phylogenetic trees were visualized with Chiplot [40].

### Bacterial isolation and growth conditions

*Vibrio splendidus* JLJ25 was isolated from the ^13^C_6_-SQ-amended microcosm derived from the JR2-PA sample, whereas *Vibrio mediterranei* AbY-1905 was previously isolated from aquaculture seawater associated with juvenile abalone [41]. Whole-genome shotgun sequencing of both strains was performed on the NovaSeq 6000 System (Illumina) using paired-end reads. The draft genomes were assembled using SPAdes [42] and annotated with PGAP through the NCBI Genome submission pipeline. Each strain was grown in modified sterile artificial seawater medium supplemented with either 3 mM SQ or 3 mM glucose as the sole carbon source. Cultures (20 ml) were grown in pre-sterilized flasks and incubated at 28°C with shaking at 160 rpm. All growth experiments were performed in independent triplicate replicates. Bacterial growth was monitored by measuring optical density at 600 nm (OD_600_) using a microplate reader (Tecan M200, Männedorf, Switzerland). Samples from each culture were collected and stored at −80°C for subsequent quantification of SQ and DHPS concentrations.

### Proteomics analysis

*Vibrio splendidus* JLJ25 or *V. mediterranei* AbY-1905 was grown in modified sterile artificial seawater medium supplemented with 3 mM SQ as the sole carbon source, whereas 3 mM glucose was added to the control treatment. Triplicate samples under each growth condition were harvested by centrifugation and digested with trypsin, as previously described [43]. The resulting peptides were desalted using SOLA SPE 96-well plates, vacuum-dried, and reconstituted in 0.1% formic acid. Peptides were separated using a Vanquish Neo UHPLC system (Thermo Scientific) and analyzed on an Orbitrap Astral mass spectrometer (Thermo Scientific) operated in data-independent acquisition (DIA) mode. Data were combined and processed using DIA-NN software [44] for database searching and protein quantification. Searches for strain AbY-1905 used the UniProt reference proteome for *Vibrio mediterranei* (taxonomy ID 689), whereas those for strain JLJ25 used the predicted proteome from the genome sequenced in this study. Search parameters were as follows: trypsin as the enzyme, a maximum of two missed cleavage sites, carbamidomethyl (C) as a fixed modification, and oxidation (M) and acetylation (protein N-terminus) as dynamic modifications. Identified proteins were filtered at a false-discovery rate of <1%. Differential expression analysis was conducted using volcano plots generated in Origin 2021, based on fold change and *P* values derived from Student’s *t-*tests for each comparison.

### Construction of *Vibrio* mutants

Scarless in-frame deletion mutants of *V. splendidus* JLJ25 (ΔACZ2GX_07095, hereafter *V. splendidus* ΔACZ2GX_07095) and *V. mediterranei* AbY-1905 (ΔAC0VPI_00125, hereafter *V. mediterranei* ΔAC0VPI_00125) were generated via homologous recombination using conjugation protocols [45]. The upstream and downstream homologous arms were amplified with specific primer pairs (Table S1) and assembled into the suicide vector pLP12 [45]. The resulting recombinant plasmid was introduced into the wild-type strain by conjugation. The first-crossover event was selected based on chloramphenicol and erythromycin resistance and confirmed by PCR, followed by induction of the second-crossover event via counter-selection. Candidate mutants were verified by PCR to confirm precise excision of the target gene.

### Genome analyses and comparative genomics

To examine the distribution of SQ degradation gene clusters across global aquatic microbiomes, 21 845 microbial genomes annotated as “aquatic_habitat” in the proGenomes database [25] were retrieved from the NCBI genome database (Table S2). Additional searches were also performed for *Vibrio* genomes (*n* = 6854, Table S3) and for reference genomes associated with ^13^C-labeled ASVs. Protein sequences (.faa) and gene positional/orientation files (.gff) for each genome were annotated using Prodigal (version 2.6.3) [46].

BLASTp searches were performed against SQ degradation cluster-associated proteins, using thresholds of ≥30% sequence identity, *E*-value ≤1 × 10^−50^, and ≥ 70% query coverage. The reference proteins (Table S4) included:

- Sulfo-EMP pathway: SQ isomerase; 6-deoxy-6-sulfofructose (SF) kinase; 6-deoxy-6-sulfofructose-1-phosphate (SFP) aldolase; 3-sulfolactaldehyde (SLA) reductase or SLA dehydrogenase.
- Sulfo-ED pathway: SQ dehydrogenase; 6-deoxy-6-sulfogluconate (SG) dehydratase; 6-deoxy-6-sulfogluconolactone (SGL) lactonase; 2-keto-3,6-dideoxy-6-sulfogluconate (KDSG) aldolase.
- Sulfo-TAL pathway: SQ isomerase; SF transaldolase; SLA reductase or SLA dehydrogenase.
- Sulfo-TK pathway: SQ isomerase; SF transketolase; 4-deoxy-4-sulfoerythrose isomerase.
- Sulfo-SMO pathway: flavin reductase, SmoA; oxidoreductase, SmoB; FMN-dependent SQ monooxygenase, SmoC.
- Sulfo-SDO pathway: SQ dioxygenase; oxidoreductase.

Candidate SQ degradation gene clusters were validated by manual inspection of gene co-localization, retaining only genomes in which all BLAST hits occurred within a 10–open reading frame window. Genomes encoding both SmoB and SmoC were designated sulfo-SMO-positive [15], whereas other SQ degradation pathways were defined by the presence of genes for all core enzymes. Genomes predicted to harbor complete SQ degradation gene clusters were taxonomically annotated using the Genome Taxonomy Database Toolkit GTDB-Tk (version 2.6.1), and phylogenetic relationships were reconstructed with IQ-TREE (version 2.0.3) [39] based on 120 bacterial marker protein-coding genes [47, 48]. The predicted proteomes of selected genomes associated with ^13^C-labeled ASVs were screened via BLASTp for protein sequences enabling catabolism of DHPS (HpsO, HpsP, and HpsN) and SL (SuyAB, SlcC, and ComC).

## Results

### Dynamics of sulfoquinovose utilization and effects on bacterial community

**Figure 1:**
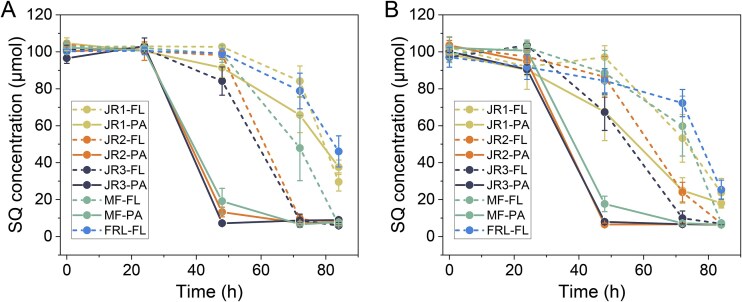
Utilization dynamics of (A) ^13^C_6_-SQ and (B) ^12^C_6_-SQ in aquatic microcosms. The time course shows that supplemented SQ was rapidly degraded by both PA (solid lines) and FL (dashed lines) bacterial communities. Sampling sites include the Jiulong River estuary (JR1, JR2, JR3), the Shenzhen Futian mangrove forest (MF), and Furong Lake (FRL). SQ, sulfoquinovose.

**Figure 2:**
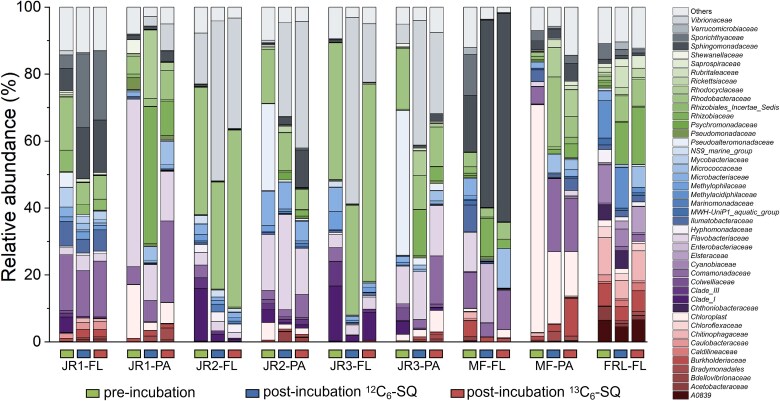
Family-level taxonomic composition of bacterial communities before and after incubation with ^12^C_6_-SQ or ^13^C_6_-SQ. Community composition was determined by 16S rRNA gene sequencing of total DNA from PA and FL communities across the Jiulong River estuary (JR1, JR2, and JR3), the Shenzhen Futian mangrove forest (MF), and Furong Lake (FRL). These profiles provide an overview of dominant bacterial families and their compositional differences before and after SQ incubation across the aquatic microcosms. Families with relative abundances <2% are grouped as “Others.” SQ, sulfoquinovose.

In all microcosm incubations, added ^13^C_6_-SQ was rapidly consumed. PA bacteria demonstrated primary consumption within 24–48 h, whereas FL bacteria exhibited a 24 h metabolic lag, with dominant assimilation occurring between 48 and 84 h (Fig. 1A). Similar temporal patterns of substrate utilization were also observed in incubations amended with ^12^C_6_-SQ (Fig. 1B). SQ consumption was accompanied by pronounced restructuring of microbial community composition (Fig. 2).

At the JR1 station, FL communities were initially dominated by *Comamonadaceae* (16.7%) and *Rhodobacteraceae* (15.8%). Following incubation with either ^12^C_6_-SQ or ^13^C_6_-SQ, *Comamonadaceae, Sphingomonadaceae*, and *Sporichthyaceae* each accounted for more than 10% of the total relative abundance. In PA communities, dominance shifted from *Flavobacteriaceae* (>50% initially) to *Comamonadaceae* (6.4% in ^12^C_6_-SQ and 24.2% in ^13^C_6_-SQ) and *Rhizobiaceae* (41% in ^12^C_6_-SQ and 10% in ^13^C_6_-SQ). At JR2, *Vibrionaceae* (primarily *Vibrio*) increased to relative abundances exceeding 15% in both FL and PA communities. *Rhodobacteraceae* remained overall stable, whereas *Cognatishimia* increased to nearly 30% in the FL community during ^13^C_6_-SQ incubation. At JR3, *Vibrionaceae* increased relative to the initial community, rising in FL fractions from <1% to 55.6% in the ^12^C_6_-SQ treatment and 17.4% in the ^13^C_6_-SQ treatment, and in PA fractions from 5.7% to over 20% in both treatments. At the MF station, the relative abundance of *Sphingomonadaceae* in FL communities increased from <17% to >55%. In contrast, chloroplast-associated sequences in PA fractions declined sharply (~70% to <22%), concurrent with ~10% increases in *Comamonadaceae* and *Rhodocyclaceae*. Finally, in the FRL-FL sample, the relative abundance of *Rhizobiaceae* increased from ~1.2% in the initial community to above 10% following incubation with either ^12^C_6_-SQ or ^13^C_6_-SQ. These shifts should be considered as descriptive community responses under SQ-amended incubation conditions and are interpreted in conjunction with the DNA-SIP results below.

### ^13^C-labeling of amplicon sequence variants during sulfoquinovose degradation

**Figure 3:**
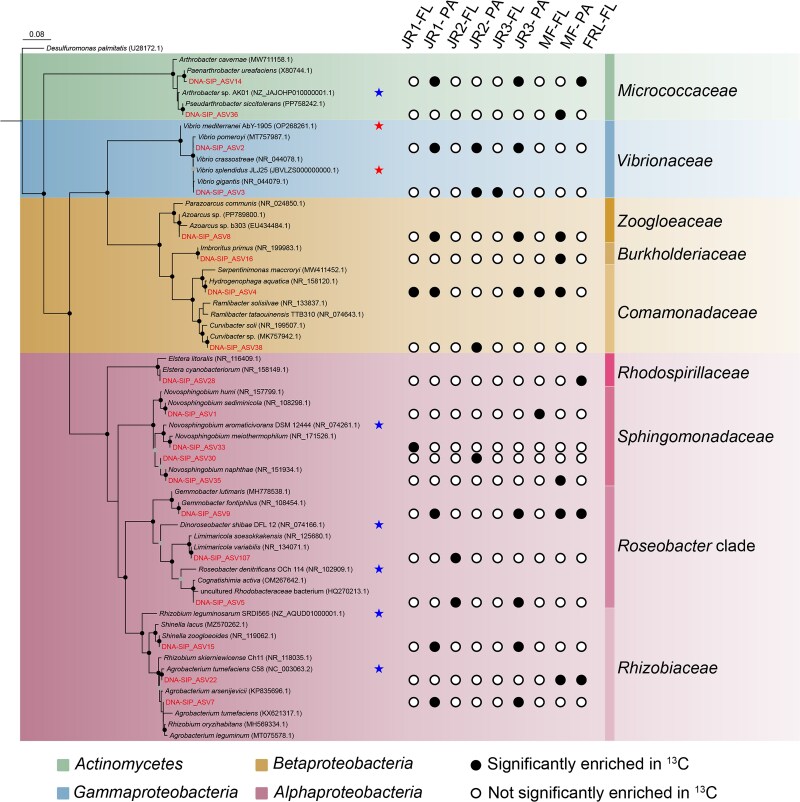
Phylogenetic analysis of 16S rRNA gene ASVs enriched in ^13^C_6_-SQ microcosms using DNA-SIP. The maximum-likelihood tree was constructed with IQ-TREE under the best-fit substitution model TIM3 + F + I + G4 and assessed using 1000 bootstrap replicates. Node support is indicated by black dots (≥90%) and grey dots (70%–90%). The scale bar shows 8% sequence divergence. 16S rRNA gene ASVs recovered in this study are highlighted in red. GenBank accession numbers are provided for the 16S rRNA gene reference sequences. Taxa that include previously reported SQ-degrading species are marked with solid stars; blue stars denote strains described in the literature, whereas red stars represent functionally validated strains from this study. Significant (Wilcoxon rank-sum tests; *P* < 0.1) ^13^C enrichment was determined by differential abundance analysis between heavy (>1.725 g ml^−1^) and light (<1.725 g ml^−1^) gradient fractions. The tree shows that ^13^C-labeled ASVs are distributed across diverse bacterial lineages, including taxa related to previously reported and newly validated SQ-degrading bacteria. SQ, sulfoquinovose.

DNA-SIP was used to identify bacterial taxa that assimilated ^13^C during growth following amendment with ^13^C_6_-SQ and therefore were associated with utilization of the amended ^13^C_6_-SQ and/or its degradation intermediates. Quantitative PCR (qPCR) across the density gradient fractions showed higher abundances of 16S rRNA genes in some heavy fractions (>1.725 g ml^−1^) in incubations with ^13^C_6_-SQ relative to the corresponding ^12^C_6_-SQ controls (Fig. S2, Table S5). These qPCR results support the incorporation of ^13^C-carbon into the community DNA. Amplicon sequencing of 16S rRNA genes was conducted across fractions from CsCl density gradients. We identified ASVs that displayed significantly higher relative abundances in heavy fractions from ^13^C_6_-SQ treatments compared with their light fractions (Fig. S3). Additionally, the peak buoyant density of 16S rRNA gene copy number shifted towards higher densities relative to the ^12^C_6_-SQ control (Fig. S4). In total, 19 ASVs were enriched in one or more samples (Fig. 3), with all exhibiting ^13^C-labeling rates exceeding 39% (Table S6).

Phylogenetic analyses of the labeled ASVs based on 16S rRNA genes identified several key taxa, including the family *Micrococcaceae* (class *Actinomycetes*), the genus *Vibrio* (class *Gammaproteobacteria*), the families *Zoogloeaceae, Burkholderiaceae*, and *Comamonadaceae* (class *Betaproteobacteria*), as well as several taxa within the *Alphaproteobacteria*, including *Rhodospirillaceae, Sphingomonadaceae, Rhizobiaceae*, and members of the *Roseobacter* clade (Fig. 3). Many of these taxa include previously characterized representatives, and the new isolates recovered in this study expand the set of cultivable representatives within these groups.

At the JR stations, 13 ASVs were significantly enriched in heavy SIP fractions from incubations with ^13^C_6_-SQ (Fig. 3). These included *Vibrio* (ASVs 2 and 3), *Zoogloeaceae* (ASV 8), *Comamonadaceae* (ASV 4 and ASV 38), *Micrococcaceae* (ASV 14), *Novosphingobium* (ASVs 30 and 33), *Shinella* (ASV 15), *Agrobacterium* (ASV 7), *Gemmobacter* (ASV 9), *Limimaricola* (ASV 107), and *Cognatishimia* (ASV 5) (Fig. 3, Fig. S3). At the MF station, eight ASVs were enriched in heavy fractions, including *Novosphingobium* (ASVs 1 and 35), *Agrobacterium* (ASV 22), *Gemmobacter* (ASV 9), *Zoogloeaceae* (ASV 8), *Comamonadaceae* (ASV 4), *Micrococcaceae* (ASV 36), and *Burkholderiaceae* (ASV 16) (Fig. 3, Fig. S3). At FRL station, four ASVs showed significant enrichment after 84 h of incubation with ^13^C_6_-SQ: *Elstera* (ASV 28), *Agrobacterium* (ASV 22), *Gemmobacter* (ASV 9), and *Micrococcaceae* (ASV 14) (Fig. 3, Fig. S3). Together, these enriched taxa represent a phylogenetically diverse assemblage of SQ-assimilating bacteria distributed across multiple aquatic stations.

We further compared the relative abundances of 16S rRNA gene-based ASVs between pre-incubation and post-incubation conditions in both ^13^C_6_-SQ- and ^12^C_6_-SQ-amended microcosms. This analysis focused on the 19 ASVs that showed enrichment in the ^13^C_6_-SQ treatments (as described above). Several ASVs increased in relative abundance between pre- and post-incubation, leading to dominance within the overall communities. In the MF-FL sample, *Novosphingobium* ASV 1 increased from <1% pre-incubation to >55% post-incubation in both ^12^C_6_-SQ- and ^13^C_6_-SQ-amended microcosms (Fig. S5). In JR2-FL *Cognatishimia* ASV 5 rose from 7.2% pre-incubation to 32.5% (^13^C_6_-SQ-amended) or 11.4% (^12^C_6_-SQ-amended) post-incubation, respectively. Similarly, *Vibrio* ASV 2 displayed variable increases across JR stations: in JR2-PA it rose from 0.2% to >10% in both ^12^C_6_-SQ- and ^13^C_6_-SQ-amended microcosms, whereas in JR3-PA it increased from 1.0% to 7.6% (^13^C_6_-SQ-amended) or 11.1% (^12^C_6_-SQ-amended) (Fig. S5). At FRL-FL, *Agrobacterium* ASV 22 increased from 0.33% to 11.7% (^13^C_6_-SQ-amended) or 6.2% (^12^C_6_-SQ-amended) (Fig. S5).

### Metabolic potential for sulfoquinovose degradation in close relatives of ^13^C-labeled amplicon sequence variants

**Figure 4:**
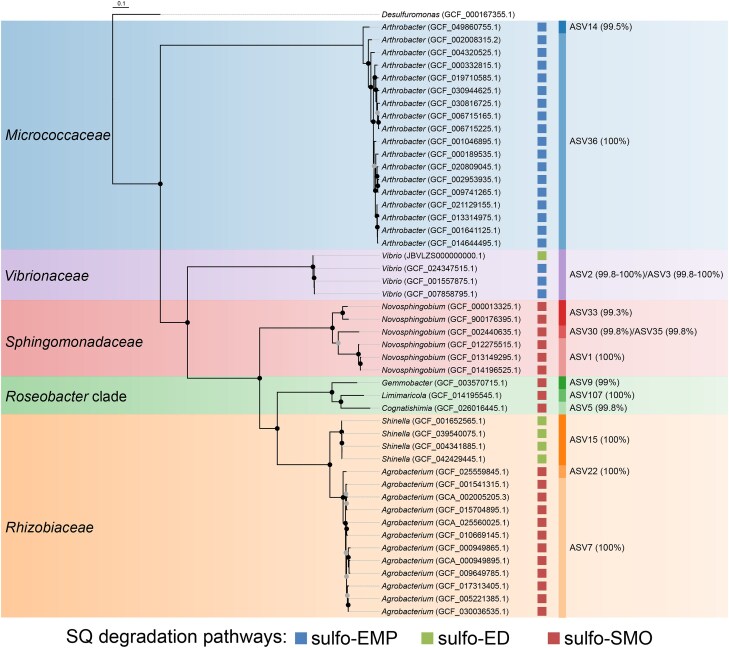
Phylogenetic analysis of genomes closely related to the ^13^C-labeled ASVs and predicted to harbor SQ degradation pathways. Based on concatenated bacterial marker genes inferred by GTDB-Tk, the maximum-likelihood tree was constructed with IQ-TREE under the best-fit substitution model LG + F + I + R4 and assessed using 1000 bootstrap replicates. Node support is indicated by black dots (≥90%) and grey dots (70%–90%). The scale bar shows 10% sequence divergence. The corresponding ^13^C-labeled ASVs are indicated on the right, with the 16S rRNA gene sequence identity between each ASV and its corresponding genome shown in parentheses. The tree shows that closely related species encode distinct SQ degradation pathways, including sulfo-EMP, sulfo-ED, and sulfo-SMO. SQ, sulfoquinovose.

To elucidate the potential pathways of SQ metabolism in the ^13^C-labeled ASVs, we examined the closest available reference genomes (Fig. 4, Table S7). Members of the *Micrococcaceae* (ASVs 14 and 36) were predicted to harbor the sulfo-EMP pathway, *Vibrio* (ASVs 2 and 3) harbored either sulfo-EMP or sulfo-ED pathways, and *Shinella* (ASV 15) was associated with the sulfo-ED pathway (Fig. 4). The sulfo-SMO pathway was predicted in multiple taxa, including *Agrobacterium* (ASVs 7 and 22), *Novosphingobium* (ASVs 1, 30, and 33), and members of the *Roseobacter* clade (ASVs 5, 9, and 107) (Fig. 4). In contrast, no canonical SQ degradation gene clusters were detected in certain taxa, such as *Zoogloeaceae* (ASV 8), *Burkholderiaceae* (ASV 16), and *Elstera* (ASV 28). Comparative genomics of their close relatives suggests that these taxa may harbor previously uncharacterized SQ degradation pathways or function as secondary degraders utilizing SQ-derived intermediates (see Discussion).

### Discovery of different sulfoquinovose degradation pathways in *Vibrio* strains

**Figure 5:**
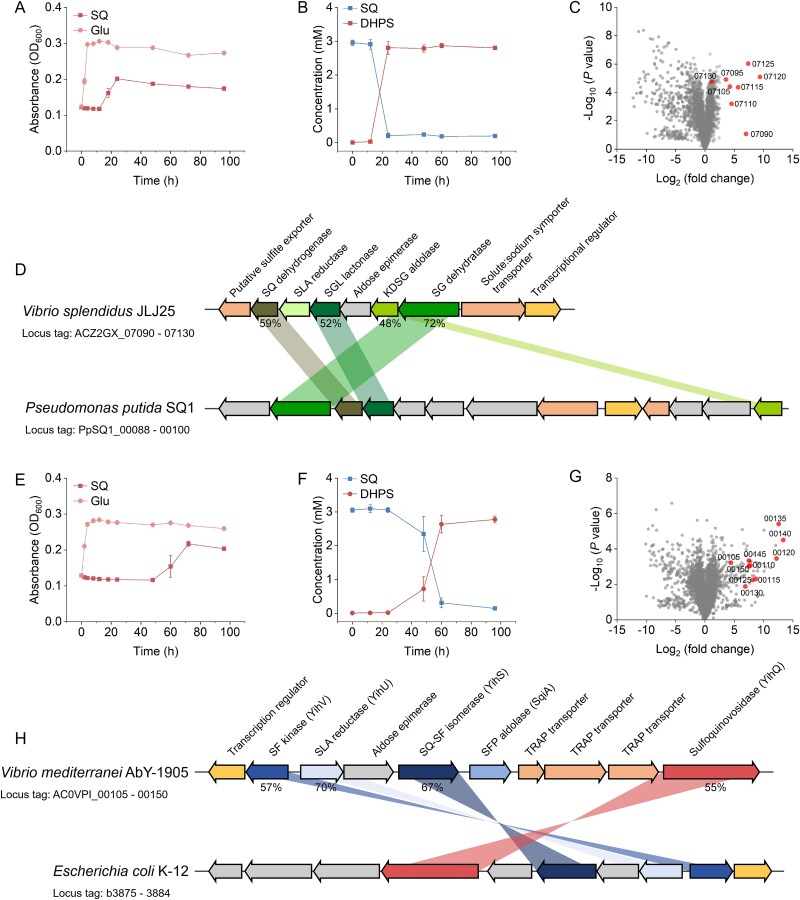
Identification of SQ degradation pathways in *Vibrio* strains. (A) Growth of *Vibrio splendidus* JLJ25 on 3 mM SQ or glucose. (B) SQ consumption and DHPS production during growth of JLJ25 on 3 mM SQ. (C) Comparative proteomic analysis of JLJ25 grown on SQ versus glucose, numbers in the figure denote locus tags with the common prefix “ACZ2GX_” omitted. (D) Gene cluster encoding the sulfo-ED pathway in JLJ25 compared to *P. putida* SQ1. (E) Growth of *Vibrio mediterranei* AbY-1905 on 3 mM SQ or glucose. (F) SQ consumption and DHPS production during growth of AbY-1905 on 3 mM SQ. (G) Comparative proteomic analysis of AbY-1905 grown on SQ versus glucose, numbers in the figure denote locus tags with the common prefix “AC0VPI_” omitted. (H) Gene cluster encoding the sulfo-EMP pathway in AbY-1905 compared to *E. coli* K-12. In both strains, growth with SQ reached approximately half of the OD_600_ observed with an equimolar concentration of glucose, and SQ consumption was accompanied by near-stoichiometric DHPS production. SQ induced the abundance of proteins encoded by sulfo-ED and sulfo-EMP gene clusters in JLJ25 and AbY-1905, respectively. The JLJ25 sulfo-ED gene cluster contains an SLA reductase instead of an SLA dehydrogenase, whereas the AbY-1905 sulfo-EMP gene cluster contains an SqiA homolog from the sulfo-EMP2 variant as the SFP aldolase. SQ, sulfoquinovose; DHPS, 2,3-dihydroxypropanesulfonate; SLA, 3-sulfolactaldehyde; SFP, 6-deoxy-6-sulfofructose-1-phosphate.

Microcosm experiments and subsequent genomic analysis of associated ^13^C-labeled ASVs indicated the potential of *Vibrio* members to degrade SQ. Therefore, we performed pure-culture experiments using SQ as the sole carbon source with two *Vibrio* isolates. *Vibrio splendidus* JLJ25 was isolated from the JR2-PA sample, and its 16S rRNA gene sequence showed 100% identity to ^13^C-labeled ASV3 and 99.77% identity to ^13^C-labeled ASV2. Under pure-culture conditions, strain JLJ25 grew robustly with SQ as the sole carbon source, converting SQ to DHPS in near-stoichiometric amounts (Fig. 5A and B). Consistent with this observation, compared to growth on an equimolar amount of glucose, the peak optical density with SQ was reduced by approximately half, indicating that only about half of the SQ carbon was assimilated for growth (Fig. 5A).

Comparative proteomics of *V. splendidus* JLJ25 grown on SQ and glucose showed that four proteins (locus tags, ACZ2GX_07095, 07105, 07115, and 07120; Fig. 5C), homologous to SQ dehydrogenase, SGL lactonase, KDSG aldolase, and SG dehydratase of the sulfo-ED pathway identified in *Pseudomonas putida* SQ1, were significantly increased in abundance (fold-change >10, *P* < 0.001, *t-*test; Fig. 5D), and which were encoded in a gene cluster. This cluster included a gene encoding a homologue of SLA reductase (31.2% similarity to YihU from *E. coli* K12 and 53.6% to SftR from *Clostridium symbiosum* LT0011), but no homolog of SLA dehydrogenase was detected. This genetic profile aligns with the observed metabolite production, where DHPS accumulated whereas SL was not detected (Fig. 5B). Knockout of the gene encoding SQ dehydrogenase (locus tag: ACZ2GX_07095) abolished growth on SQ, whereas it had no effect on growth with glucose (Fig. S6A). Taken together, these data support the conclusion that *V. splendidus* JLJ25 degrades SQ via a modified sulfo-ED pathway terminating in DHPS rather than SL.

In addition to strain JLJ25, a strain of *V. mediterranei* AbY-1905, previously isolated from the aquaculture area of Sanggou Bay in China [41], could also utilize SQ as the sole carbon source for growth and produced a stoichiometric amount of DHPS in single carbon source culture (Fig. 5E and F). Comparative proteomics showed that growth on SQ significantly increased the abundance of proteins encoded by a cluster of 10 genes (locus tags: AC0VPI_00105 to AC0VPI_00150; fold-change >20, *P* < 0.05, *t*-test) (Fig. 5G). The proteins encoded in this 10-gene cluster share high homology with key enzymes of the sulfo-EMP pathway from *E. coli* K-12. However, this gene cluster contains an SqiA homologue from the sulfo-EMP2 variant pathway as the SFP aldolase, rather than YihT. Consistently, a mutant strain lacking the gene for SQ isomerase (locus tag: AC0VPI_00125) lost its ability to degrade SQ (Fig. S6B). These findings demonstrate that this strain degrades SQ via a hybrid sulfo-EMP pathway (Fig. 5H).

**Figure 6:**
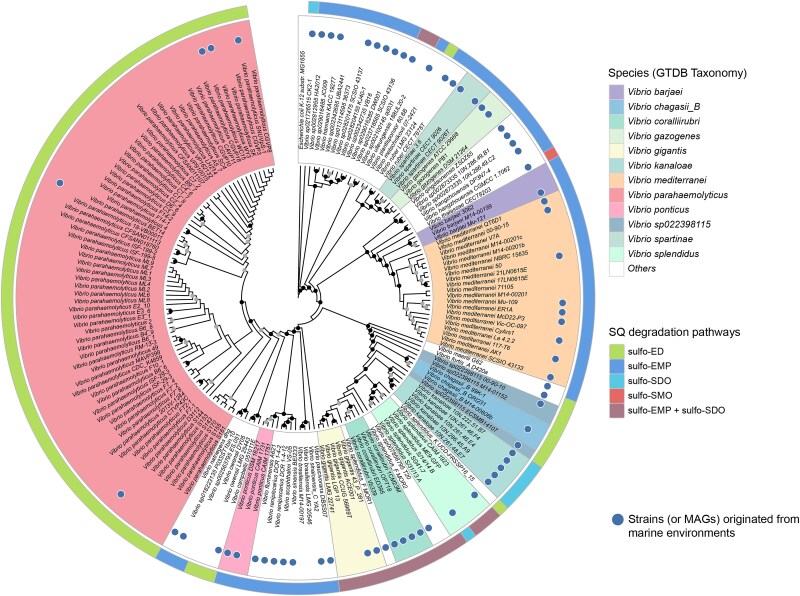
Diversity of SQ degradation pathways within the genus *Vibrio*. Based on concatenated bacterial marker genes inferred with GTDB-Tk, the maximum-likelihood tree was constructed with IQ-TREE under the best-fit substitution model Q.YEAST+F + I + R5 and assessed using 1000 bootstrap replicates. Node support is indicated by black dots (≥90%) and grey dots (70%–90%). Putative SQ degradation pathways, including sulfo-EMP, sulfo-ED, sulfo-SMO, and sulfo-SDO, are shown in different colors. In total, 170 genomes contained candidate SQ degradation pathways. Several *Vibrio* taxa encoded both sulfo-EMP and sulfo-SDO pathways. SQ, sulfoquinovose.

The two different SQ degradation pathways identified within these *Vibrio* strains prompted a genus-wide survey of SQ metabolic potential. We analyzed 6854 *Vibrio* genomes retrieved from RefSeq and GenBank (Table S3) and identified candidates containing four putative SQ degradation pathways—sulfo-EMP, sulfo-ED, sulfo-SMO, and sulfo-SDO—in 2.5% (*n* = 170) of the genomes (Table S8). The same hybrid sulfo-EMP/EMP2 pathway was present in 81 genomes spanning *V. mediterranei, V. gigantis, V. coralliirubri, V. splendidus*, and 34 additional *Vibrio* species, representing the greatest species-level diversity of SQ degradation pathways within the genus (Fig. 6; Table S8). The sulfo-SDO pathway was detected in 25 genomes distributed across nine *Vibrio* species, and several taxa (e.g. *V. gigantis, V. coralliirubri, V. splendidus, V. splendidus* F, *V. ulleungensis, Vibrio* sp002100145, and *Vibrio* sp007858795) encoded both sulfo-SDO and sulfo-EMP pathways.

The sulfo-ED pathway was also identified in 81 genomes but showed strong phylogenetic clustering, with the majority (69/81) confined to *Vibrio parahaemolyticus*, and the remaining genomes were distributed among seven other species (Fig. 6). All sulfo-ED pathways were predicted to produce DHPS as the end-product of sulfoglycolysis. In contrast, the sulfo-SMO pathway was detected in only a single genome, namely *Vibrio thalassae* (Fig. 6).

From a biogeographic perspective, nearly 40% of *Vibrio* strains harboring SQ degradation pathways were isolated from marine environments (Fig. 6), and these strains were frequently associated with a host-associated (e.g. *Magallana gigas*) lifestyle (Table S8), suggesting an ecological role of SQ utilization in marine and host-associated habitats.

### Diverse sulfoquinovose degradation pathways in global aquatic bacterial genomes

**Figure 7:**
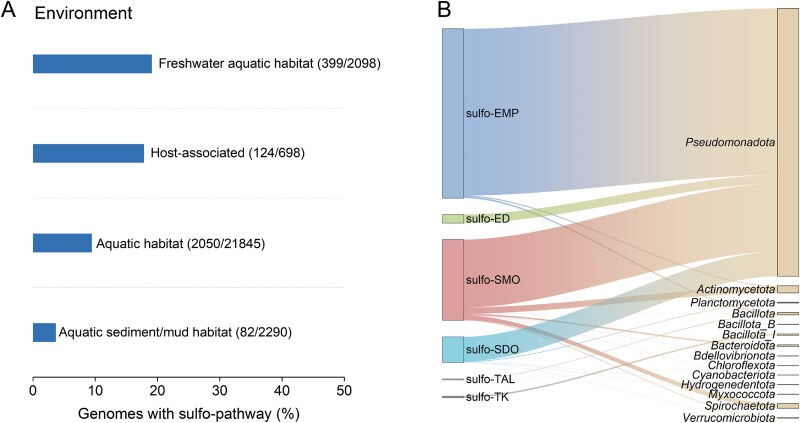
Prevalence and taxonomic distribution of SQ degradation pathways in aquatic bacterial genomes from proGenomes. (A) Prevalence of SQ degradation pathways, including sulfo-EMP, sulfo-ED, sulfo-SMO, sulfo-SDO, sulfo-TAL, and sulfo-TK, across different aquatic environments. Bars indicate the percentage of genomes containing at least one predicted SQ degradation pathway, and numbers in parentheses denote the count of pathway-containing genomes relative to the total number of genomes analyzed in each environment. (B) Phylum-level taxonomy of bacterial genomes containing SQ degradation pathways, classified according to GTDB taxonomy. Flows connect individual SQ degradation pathways to bacterial phyla, with flow width proportional to the number of genomes. SQ, sulfoquinovose.

To evaluate the global prevalence of the six previously described SQ degradation pathways, we conducted BLASTp searches against 21 845 microbial genomes derived from global aquatic habitats. SQ degradation pathways were predicted in 2050 genomes (Table S9), representing over 9% of the total (Fig. 7A). Prevalence varied among different habitats: freshwater genomes exhibited SQ degradation pathway frequencies that were fivefold higher than those in sediment habitats (Fig. 7A). Beyond clades already recognized as rich in SQ pathways, such as *Enterobacterales, Rhodobacterales*, and *Rhizobiales* within the *Pseudomonadota*, we also detected frequent encoding of sulfo-SMO gene clusters in *Actinomycetota* and *Spirochaetota* (Fig. 7B). These findings indicate that SQ degradation pathways are broadly distributed across diverse bacterial lineages and habitats. Their widespread presence suggests that bacterial SQ catabolism is a common metabolic capability in aquatic ecosystems, with likely contributions to global carbon and sulfur cycling. The prominence of sulfo-SMO clusters in these genomes aligns with the dominance of sulfo-SMO–associated taxa in our DNA-SIP experiments, supporting a correspondence between genomic potential and SQ utilization under the incubation conditions used here.

## Discussion

In this study we employed a culture-independent approach, DNA-SIP, to identify microbial taxa actively involved in degrading SQ across three diverse aquatic environments. By tracing the selective incorporation of ^13^C from ^13^C_6_-SQ into DNA, we show that SQ metabolism is a common community trait, i.e. carried out by a specialized subset of microorganisms. Combining SIP with physiological assays of cultured isolates and comparative proteomics, we directly confirmed SQ utilization and linked genetic potential to metabolic activity, supporting the metabolic potential inferred from SIP. This approach strengthens ecological interpretations and acknowledges the limitations of SIP in fully resolving complex cross-feeding dynamics or capturing activity from rare or slow-growing taxa.

Within marine systems, members of the genus *Vibrio* particularly stood out in this study, as they were labeled in DNA-SIP analyses across several samples (Fig. 3). The capacity of closely related *Vibrio* isolates to utilize SQ is supported by the presence of complete and expressed gene clusters that encode key components of the enzymatic machinery required for SQ transport and intracellular metabolism (Fig. 5C, D, G, and H). These results provide direct experimental evidence for SQ utilization in *Vibrio* and highlight the value of combining isolate physiology with SIP observations. The genomic organization of these clusters is reminiscent of well-studied polysaccharide utilization loci [49], suggesting that SQ degradation represents a strategy for efficiently accessing this abundant organosulfur resource. Whereas previous research has emphasized the roles of the *Roseobacter* clade and *Alteromonas* as key organosulfur degraders in the phycosphere [3, 6], our data reveal that metabolically versatile *Vibrio* species are also major contributors to SQ turnover in coastal waters. Both isolates produced DHPS as the end product of sulfoglycolysis. DHPS is a prominent organosulfur metabolite in marine systems and is produced by various algae [50, 51]. Our findings suggest that *Vibrio*-mediated SQ sulfoglycolysis may therefore contribute to the marine DHPS pool.

Further analysis revealed metabolic diversity within the genus *Vibrio*. Comparative proteomic analysis and systematic genomic analyses indicate that *Vibrio* lineages have acquired distinct strategies for SQ metabolism. Even closely related strains employ different pathways (Fig. 6), underscoring metabolic differentiation within the genus. Such variation may confer flexibility in metabolism under different environmental conditions, although its ecological significance *in situ* remains to be quantified. *Vibrio* species carrying SQ degradation genes are often associated with diverse marine hosts, including oysters, clams, and corals [52, 53] (Table S8), indicating that SQ degradation may occur within host-microbe systems. The strain AbY-1905 in this study was isolated from aquaculture seawater associated with juvenile abalone [41], further supporting the potential role of *Vibrio* in SQ degradation in host-associated niches. Given that *Vibrio* species are widespread and abundant members of estuarine and marine microbial communities [54–57], and can metabolize other major marine organosulfur compounds such as dimethylsulfoniopropionate and dimethyl sulfide [58], these findings underscore their broad involvement in organic sulfur catabolism and deepen our understanding of *Vibrio* ecological physiology.

Within freshwater SQ incubations, *Novosphingobium* and *Agrobacterium* emerged as the predominant SQ degraders and these genera included genomes predicted to encode the sulfo-SMO pathway. The identification of these taxa validates the SIP results, as some species within these genera have been experimentally validated to use SQ as a sole carbon source via the sulfo-SMO pathway, and their enzymatic mechanisms have been elucidated [14, 15]. This pathway enables complete assimilation of the six-carbon backbone of SQ and releases sulfite or sulfate for bacterial assimilation [6]. *In situ* measurements revealed that SQ concentrations in freshwater are about one order of magnitude higher than in seawater (Fig. S7, Table S10). Given the substantial input of terrestrial plant litter, the quantity of SQ entering many freshwater ecosystems is likely to be substantial, suggesting that SQ is an important organic sulfur reservoir in these systems. The prevalence of SQ degradation gene clusters in freshwater microbial genomes (~15%) is higher than the overall average for aquatic environments (<10%) (Fig. 7A), indicating that heterotrophic SQ metabolism may represent an important component of freshwater sulfur cycling.

Biogeography reveals distinct ecological niches of the *Novosphingobium* and *Agrobacterium* genera. Approximately 24% of *Novosphingobium* genomes harboring SQ degradation potential were recovered from freshwater habitats, with additional occurrences in soils and sediments (Fig. S8; Table S11), indicating a broad distribution of this trait across terrestrial and aquatic ecosystems. Most *Agrobacterium* genomes encoding the sulfo-SMO pathway are primarily plant-associated (Fig. S9; Table S12), consistent with the abundance of plant-derived SQ in photosynthetic membranes. The detection of plant-associated *Agrobacterium* in lake ecosystems may reflect terrestrial plant inputs into these freshwater systems. Together, these findings indicate that distinct microbial taxa dominate SQ degradation in marine versus freshwater ecosystems.

Beyond the well-characterized primary degraders, our SIP data point to a wider cast of SQ-utilizing bacteria. Several ^13^C-labeled taxa do not carry the standard SQ degradation gene clusters. They may therefore use unknown SQ pathways, or they may feed on metabolites released by other bacteria. For instance, the ^13^C-labeled ASV 28, assigned to *Elstera*. Related genomes encode a GH31 sulfoquinovosidase (Fig. S10A), a gateway enzyme for SQ degradation [9], as well as a partial sulfo-ED pathway. However, these gene clusters lack an obvious SGL lactonase, which is normally required in the canonical sulfo-ED pathway. This suggests that *Elstera* may use a non-canonical version of the pathway that bypasses this step, perhaps resembling De Ley–Doudoroff-type variants of the Entner-Doudoroff pathway [59, 60]. Other labeled taxa appear to be secondary degraders. *Zoogloeaceae* ASV 8 and *Burkholderiaceae* ASV 16 are identical by 16S rRNA sequence, to species that encode pathways for DHPS and SL degradation (Fig. S10B). This supports a role in cross-feeding on sulfoglycolysis products. These intermediates can arise from different primary degraders. *Arthrobacter* sp. AK01 (*Micrococcaceae*) produces SL via the sulfo-EMP pathway [21], whereas *P. putida* SQ1, isolated from freshwater lake sediment, produces SL through the sulfo-ED pathway [12]. By contrast, our *Vibrio* isolates produce DHPS through sulfo-ED or sulfo-EMP pathways (Fig. 5). Together, these findings suggest that SQ degradation in environmental communities is not a solo process. It may instead depend on a network of primary degraders and secondary consumers. This cross-feeding could improve community-level use of SQ-derived carbon and sulfur, much like the cooperative breakdown of extracellular DNA in bacterial communities [61]. It also raises the possibility that sulfoglycolytic bacteria act as keystone species supporting specialist consumers of short-chain organosulfonates.

In conclusion, supplementation of marine and freshwater microcosms with SQ results in its rapid uptake (Fig. 1), revealing a substantial and widespread latent capacity for SQ utilization across aquatic microbial communities. By coupling this community-level response with DNA-SIP, isolate physiology and comparative proteomics (Fig. 5A, C, E, and G), and genomic analysis, we show that this uptake is mediated by a phylogenetically diverse set of bacteria encoding multiple SQ catabolic strategies across several habitats (Fig. 7). These data provide direct experimental evidence that SQ degradation is an active and widespread process in aquatic ecosystems, supporting its importance as a microbial source of carbon and possibly, sulfur. Our results suggest that SQ turnover in these environments involves both primary degraders and cross-feeding to secondary degraders that complete organosulfur biomineralization. Although SQ concentrations and metabolism in marine environments have begun to be defined on a global scale [3], its dynamics and reservoirs in freshwater environments remain much less well understood. Future research should therefore quantify SQ fluxes across aquatic habitats by integrating metatranscriptomics, metagenome-centered analyses, and *in situ* metabolite tracing to link genetic potential with pathway expression, and track the fate of SQ-derived carbon and sulfur in microbial biomass and the broader organic matter pool, thereby linking microbial metabolism to ecosystem-scale biogeochemical function.

## Supplementary Material

Supplementary_material_wrag155

## Data Availability

The raw sequencing data of bacterial 16S rRNA genes are available in the NCBI database under accession number PRJNA1438631 (https://www.ncbi.nlm.nih.gov/bioproject/PRJNA1438631). The genome sequence data generated in this study have been deposited in the NCBI database under accession numbers PRJNA1437828 (https://www.ncbi.nlm.nih.gov/bioproject/PRJNA1437828) for strain *Vibrio mediterranei* AbY-1905 and PRJNA1426928 (https://www.ncbi.nlm.nih.gov/bioproject/PRJNA1426928) for strain *Vibrio splendidus* JLJ25. The proteomic data generated in this study have been deposited in the ProteomeXchange Consortium (https://proteomecentral.proteomexchange.org) via the iProX partner repository under accession number PXD075831 (https://proteomecentral.proteomexchange.org/?pxid=PXD075831).
