# Supplementary material for: Widespread marine and freshwater distributions of active sulfoquinovose-degrading bacteria
Source: ISME J. 2026 Jun 17;20(1):wrag155. doi: 10.1093/ismejo/wrag155 (PMC13372026; doi:10.1093/ismejo/wrag155)
Supplement: Supplementary_material_wrag155 [file supplementary_material_wrag155.zip › Supplementary_Figures_wrag155.pdf]

Supplementary Figures for

Widespread marine and freshwater distributions of active sulfoquinovose-degrading bacteria

Guohua Liu[1,#], Xuanyun Qiu[1,#], Rongguang Cao[1], Changjie Dong[1], Quanrui Chen[1], Shujing Liu[1], Wenhao Li[1], Xuejing Li[1], Nianzhi Jiao[1], Spencer J. Williams[2], Yao Zhang[1], Kai Tang[1,*]

*Address correspondence to: Kai Tang, State Key Laboratory of Marine Environmental Science, Fujian Key Laboratory of Marine Carbon Sequestration, College of Ocean and Earth Sciences, Xiamen University, Xiang'an South Road, Xiamen 361102, China. Email: tangkai@xmu.edu.cn

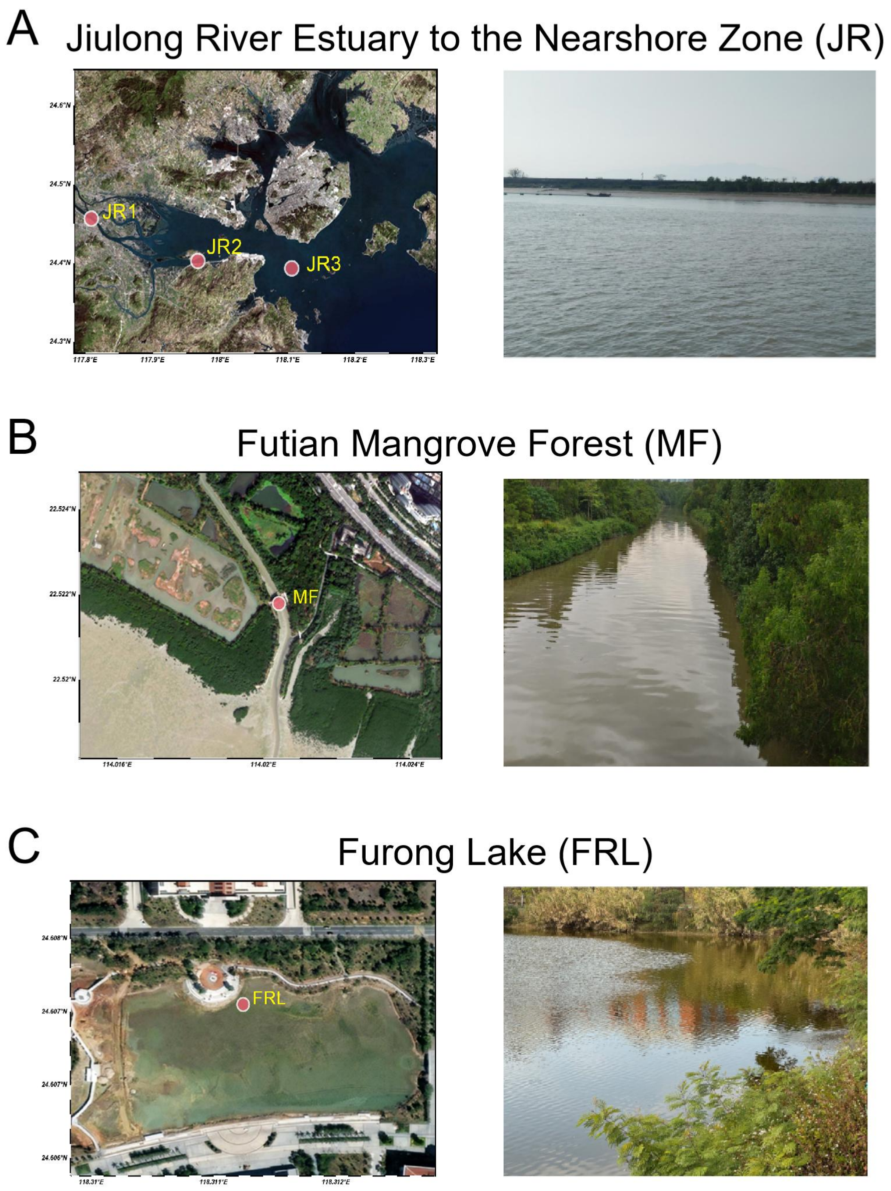

Figure S1. Geographic locations and geochemical characteristics of sampling stations. (A) Jiulong River estuary (stations JR1, JR2, JR3). (B) Shenzhen Futian mangrove forest (MF). (C) Furong Lake (FRL). For each station, key geochemical parameters were measured, including temperature, salinity, pH, and SQ concentration (Table S10).

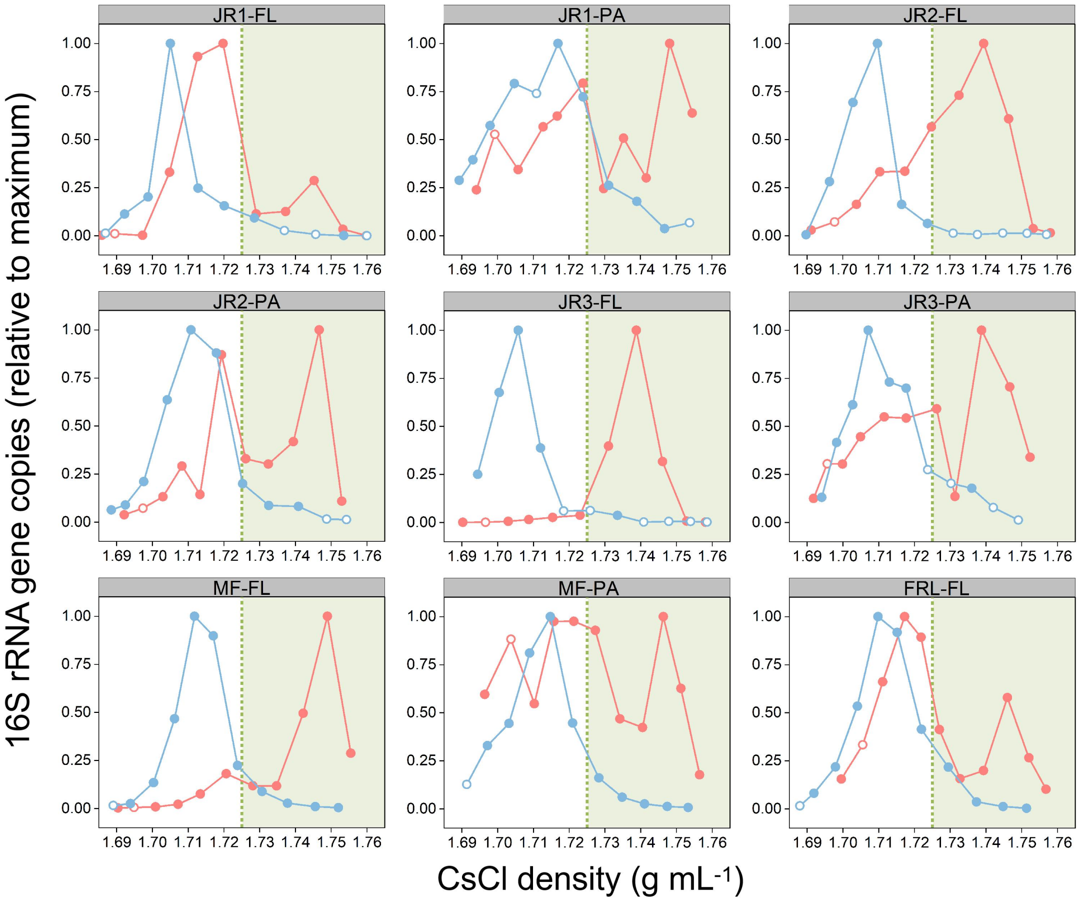

Figure S2. Proportions of 16S rRNA gene copies recovered from fractions of DNA-SIP gradients across microcosm incubations. Distributions are shown for microcosm incubations from sampling stations with $^{12}C_6$-SQ (blue traces) and $^{13}C_6$-SQ (red traces). Filled symbols indicate that DNA from the respective fraction of the DNA-SIP gradient was used as a template for amplicon sequencing. The green shaded area indicates the density range of the gradient above which ^{13}C-labeled DNA is expected to accumulate. FL, free-living bacterial communities; PA, particle-associated bacterial communities.

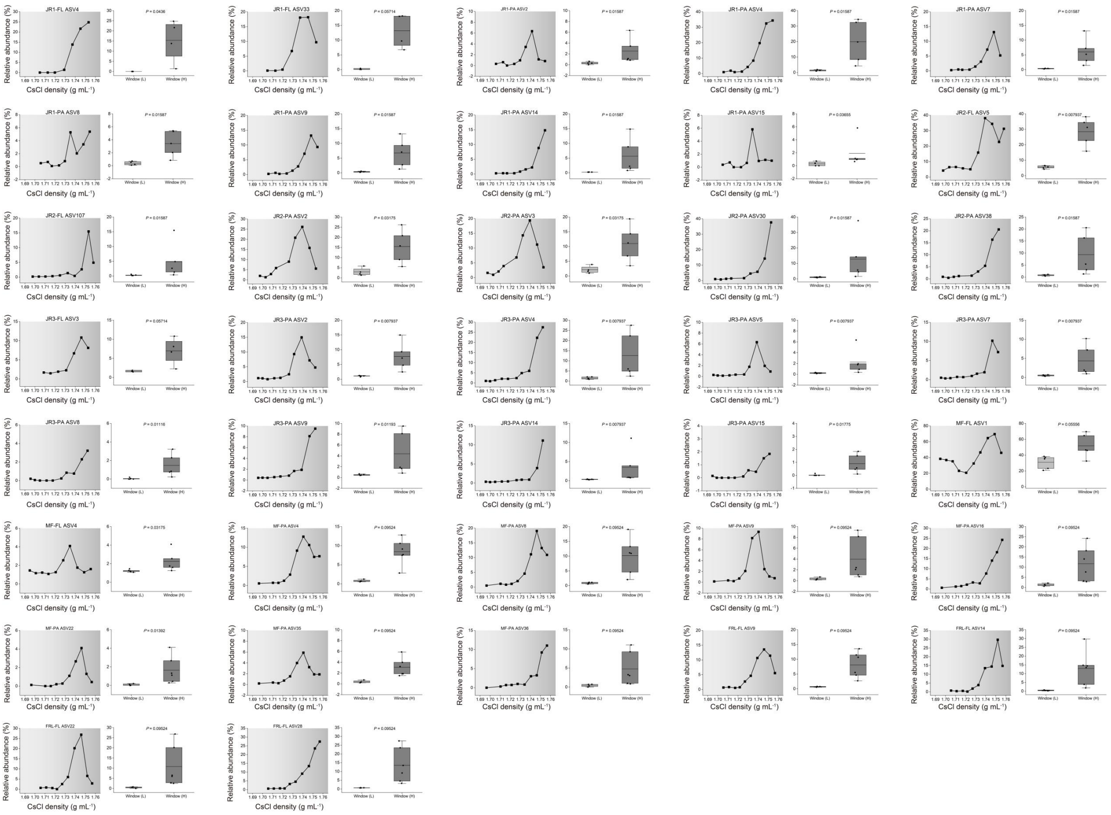

Figure S3. DNA-SIP reveals ^{13}C-carbon uptake from $^{13}C_6$-SQ by specific taxa. Line plots show the relative abundances of ASVs across DNA-SIP density fractions after incubation with $^{13}C_6$-SQ. The gray shaded area indicates the denser “heavy” end of the gradient. Accompanying box plots compare relative abundances between heavy (Window (H), >1.725 g mL^{-1}) and light (Window (L), <1.725 g mL^{-1}) gradient fractions. In the box plots, the center line represents the mean; box limits represent the 25th and 75th percentiles; whiskers extend 1.5 times the interquartile range from the 25th and 75th percentiles. Statistical significance of differences between heavy and light fractions was assessed using a Wilcoxon rank-sum test in R, and corresponding *P* values are reported.

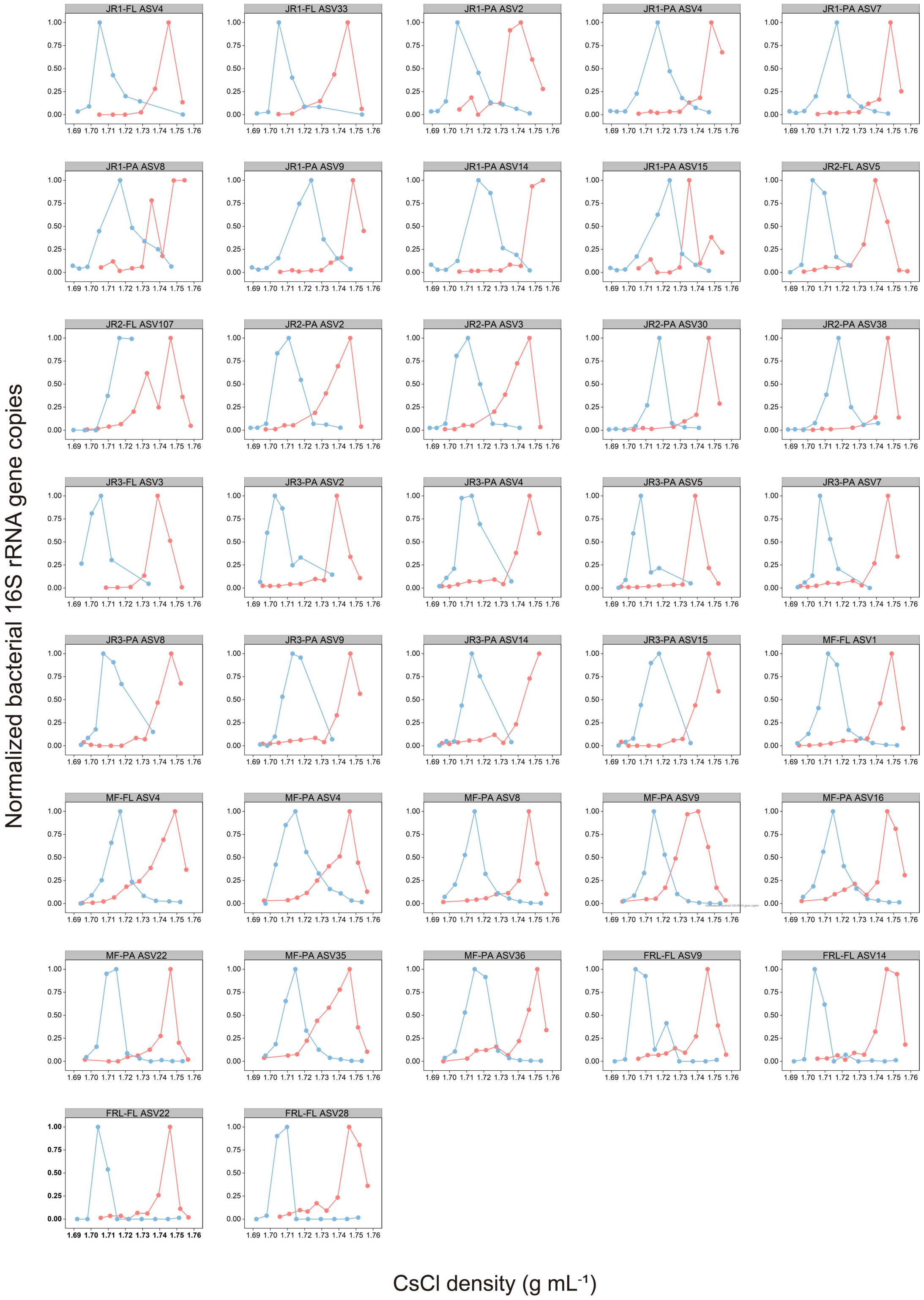

Figure S4. Normalized distribution of bacterial 16S rRNA gene copies of ^{13}C-labeled ASVs across CsCl density gradients. Distributions are shown for microcosm incubations with $^{12}C_6$-SQ (blue traces) and $^{13}C_6$-SQ (red traces). Data are normalized to values between 0 and 1 along each gradient. FL, free-living bacterial communities; PA, particle-associated bacterial communities.

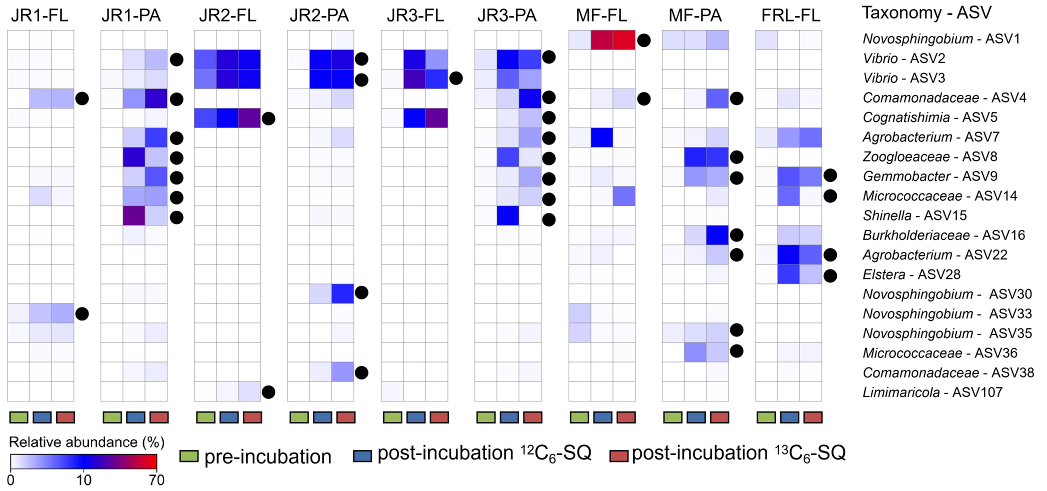

Figure S5. Heatmaps display the relative sequence abundances of each ^{13}C-labeled ASV in the total microbial community before and after incubation with $^{12}C_6$-SQ or $^{13}C_6$-SQ, based on non-fractionated 16S rRNA gene amplicon sequencing. ASVs identified as ^{13}C-labeled are indicated by a filled black circle.

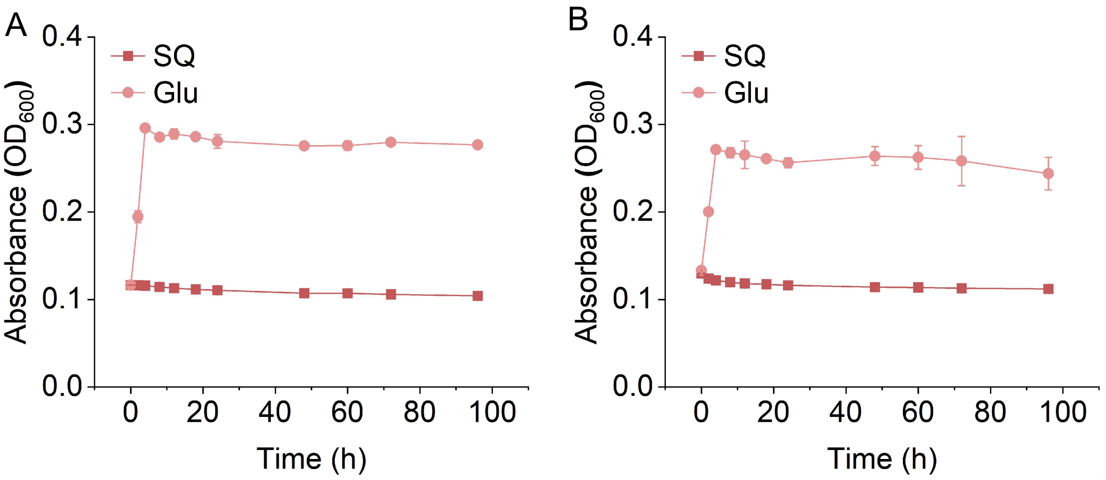

Figure S6. Growth of (A) *Vibrio splendidus* JLJ25 ΔACZ2GX_07095 and (B) *Vibrio mediterranei* AbY-1905 ΔAC0VPI_00125 mutants on SQ and glucose.

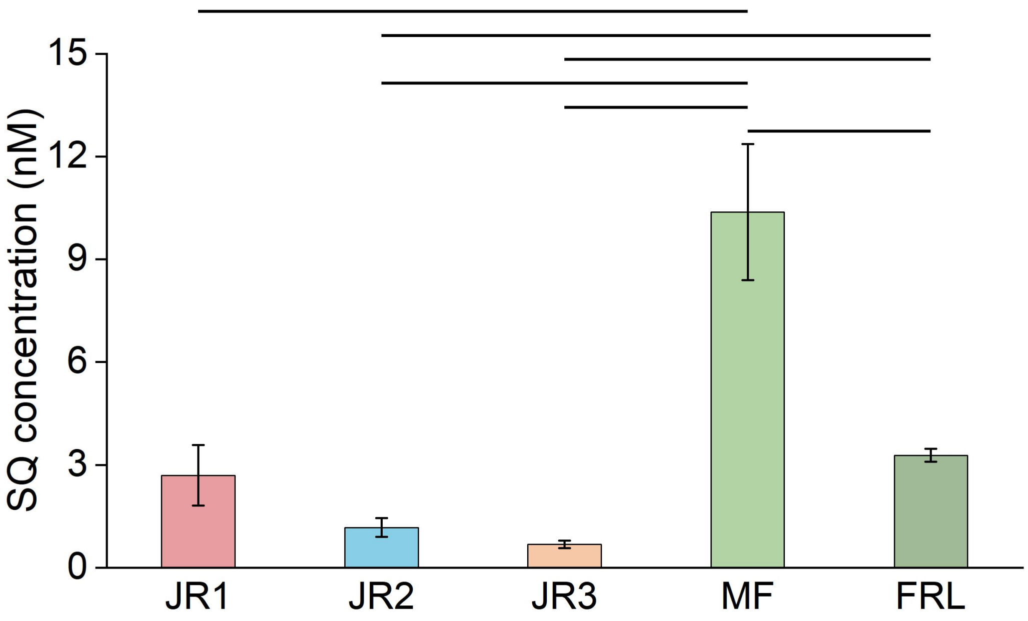

Figure S7. Concentrations of SQ across sampling stations. Error bars represent the standard deviation (n = 3). Black lines indicate significant differences in SQ concentrations between sampling sites (*t*-test; $P < 0.05$).

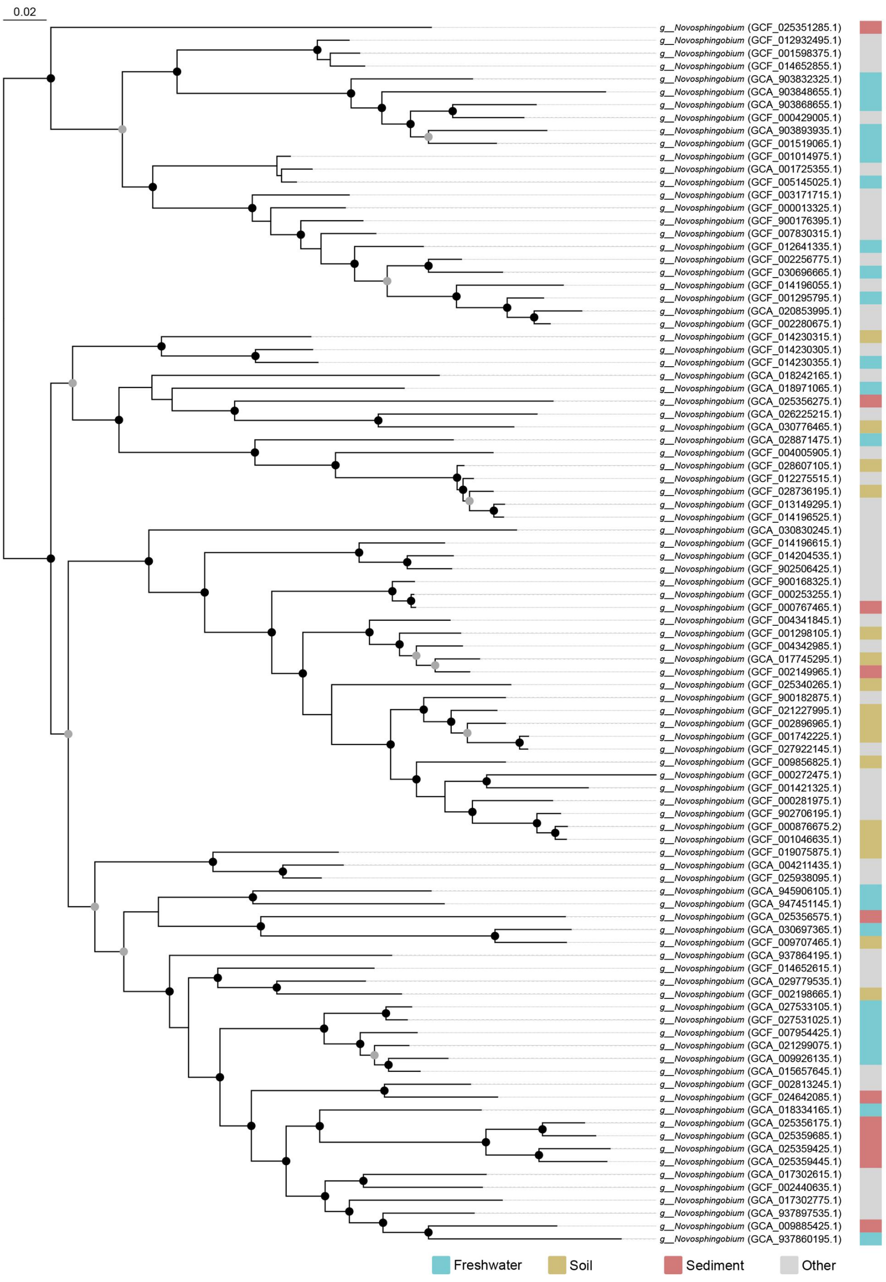

Figure S8. Phylogenetic distribution of predicted sulfo-SMO pathway among *Novosphingobium* genomes retrieved from the GTDB release220 reference database. Based on concatenated bacterial marker genes inferred with GTDB-Tk, the maximum-likelihood tree was constructed with IQ-TREE under the best-fit substitution model Q.INSECT+F+R7 and assessed using 1,000 bootstrap replicates.Node support is indicated by black dots (≥90%) and grey dots (70–90%). The scale bar shows 2% sequence divergence. These organisms originate from diverse habitats, including freshwater, soil, sediment, and other environments.

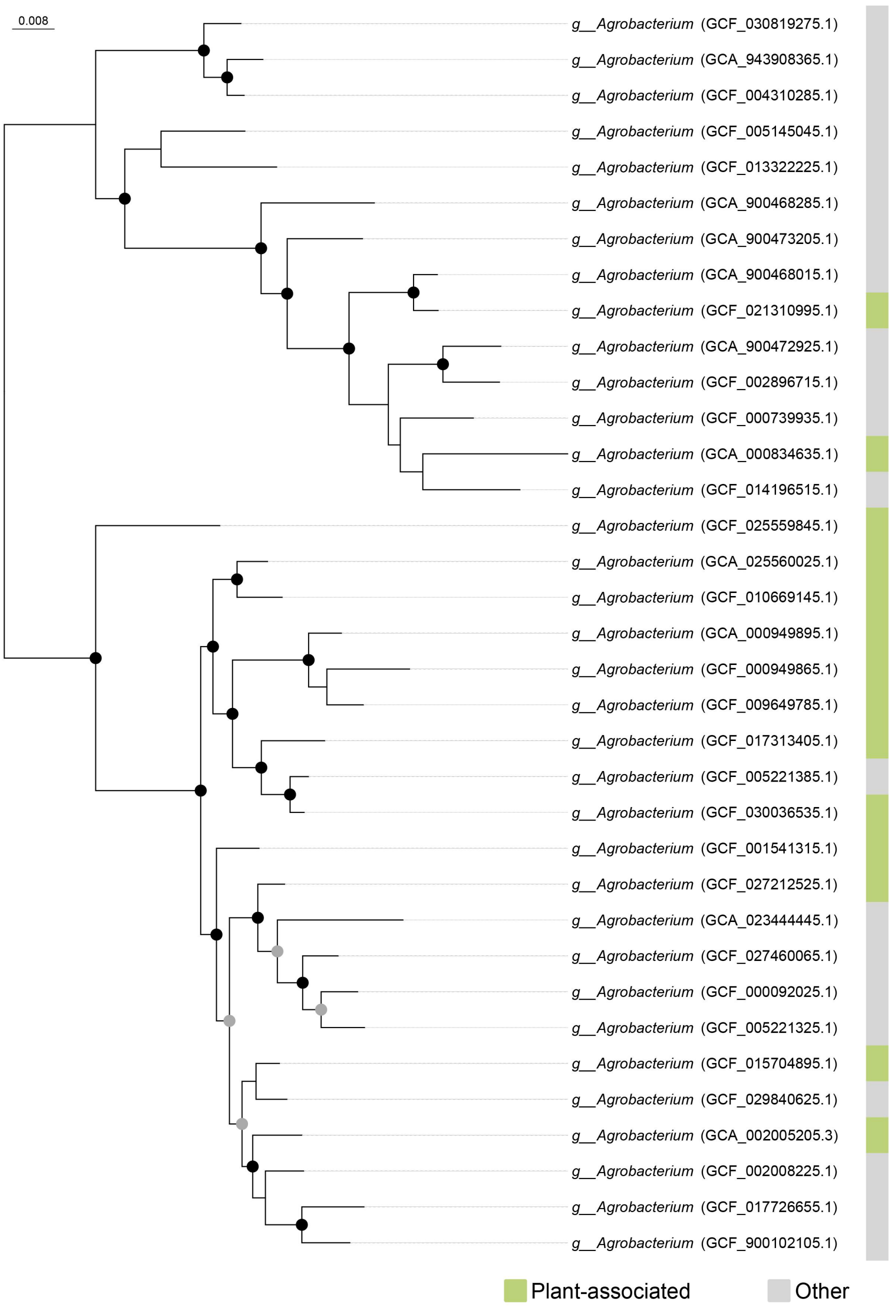

Figure S9. Phylogenetic distribution of predicted sulfo-SMO pathway among *Agrobacterium* genomes retrieved from the GTDB release220 reference database. Based on concatenated bacterial marker genes inferred with GTDB-Tk, the maximum-likelihood tree was constructed with IQ-TREE under the best-fit substitution model Q.INSECT+F+I+R3 and assessed using 1,000 bootstrap replicates. Node support is indicated by black dots (≥90%) and grey dots (70–90%). The scale bar shows 0.8% sequence divergence. The habitats of these organisms include plant-associated and other environments.

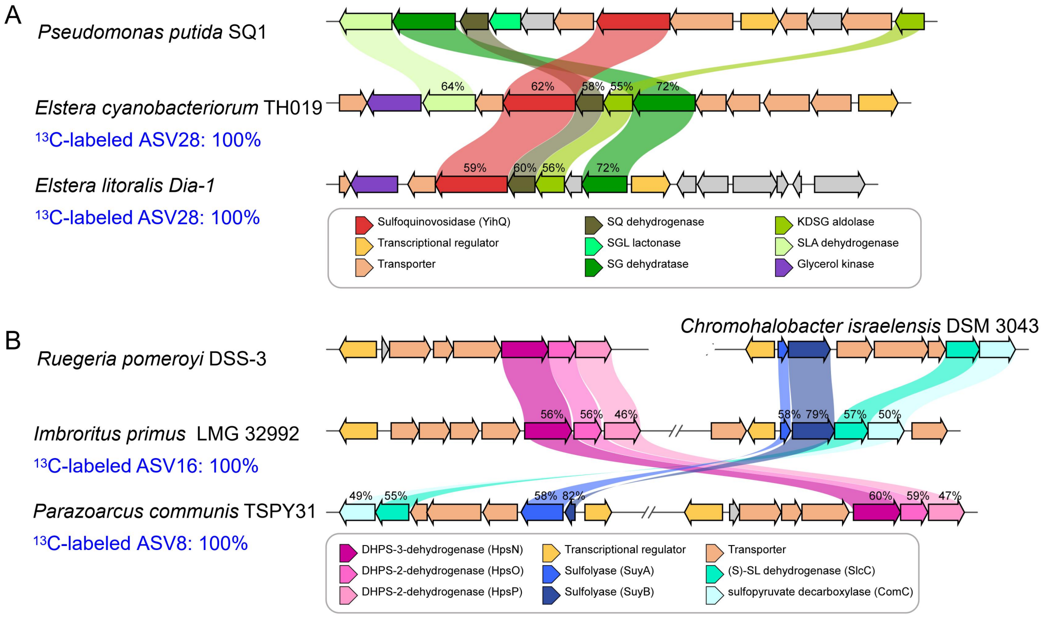

Figure S10. Genomes closely related to ^{13}C-labeled ASVs reveal potential previously uncharacterized SQ degradation pathways and cross-feeding. (A) Genomes associated with ^{13}C-labeled ASV28 encode a predicted GH31 family sulfoquinovosidase (YihQ) but lack complete SQ degradation gene clusters. (B) Genomes associated with ^{13}C-labeled ASV16 and ASV8 contain predicted gene clusters for degradation of secondary SQ metabolites DHPS and SL. Percentages in blue indicate 16S rRNA gene sequence identity between each ASV and the corresponding genome. Percentages within gene clusters indicate amino acid sequence identity to the corresponding reference proteins. *Pseudomonas putida* SQ1, *Ruegeria pomeroyi* DSS-3, and *Chromohalobacter israelensis* DSM 3043 denote previously characterized strains [1–3].

Supplementary references

1. Felux A-K, Spiteller D, Klebensberger J *et al*. Entner-Doudoroff pathway for sulfoquinovose degradation in *Pseudomonas putida* SQ1. *Proc Natl Acad Sci USA* 2015;**112**:E4298-E4305

2. Liu L, Gao X, Dong C *et al*. Enantioselective transformation of phytoplankton-derived dihydroxypropanesulfonate by marine bacteria. *ISME J* 2024;**18**:wrae084.

3. Denger K, Cook AM. Racemase activity effected by two dehydrogenases in

sulfolactate degradation by Chromohalobacter salexigens: purification of (S)-sulfolactate dehydrogenase. *Microbiology* 2010;**156**:967-974.
